# Enhanced therapeutic efficacy of *Eupolyphaga sinensis* Walker in females through sex-specific metabolomic-pharmacodynamic divergence

**DOI:** 10.1038/s41598-025-90100-5

**Published:** 2025-02-19

**Authors:** Chenghao Fei, Jie Zou, Zhaorui Yang, Huaiyang Chang, Lixian Lu, Kun Zhao, Hongzhuan Shi

**Affiliations:** Institute of Chinese Medicinal Materials, Nanjing Agricultural University, Nanjing, 210095 Jiangsu Province People’s Republic of China

**Keywords:** *Eupolyphaga sinensis* Walker, Traditional medicine, Sex differences, Metabolomics, Thrombosis, Osteoporosis, Medicinal chemistry, Pharmacology

## Abstract

*Eupolyphaga sinensis* Walker (ESW), a medicinal insect used in traditional Chinese medicine, is renowned for its effects on blood circulation, stasis resolution, and bone and tendon healing. The underlying reasons for the clinical preference for female ESW remain unclear. Previous investigations were limited in scope, focusing narrowly on female specimens, large-molecule compounds, and single pharmacological effect. This study systematically compared female and male ESW in terms of composition and therapeutic efficacy. Metabolomics identified 31 compound types in both female and male ESW, including lipids, amino acids, and fatty acids. Female ESW exhibited significantly higher levels of 8 bioactive compounds, 15 small peptides, and 13 prostaglandins compared to male ESW, which contribute to immunity enhancement, antithrombotic effects, and improved bone metabolism. These differences may underlie the superior medicinal efficacy of female ESW. In the thrombosis model, ESW can cause vasodilation, reduce blood cell aggregation and thrombosis rate of mice tails. It also improved t-PA levels, prolonged APTT, and enhanced hepatic SOD activity, with female ESW showing stronger effects on MDA and D2D levels, indicating its stronger ability to protect cells from damage and fibrinolytic effect. In the osteoporosis model, ESW increased femur length, liver, and thymus indices while regulating serum BALP and Mg levels. Female ESW notably reduced TRACP-5b, OT/BGP, P, and Cu to normal levels, indicating its stronger ability to improved bone metabolism, corrected disturbances in calcium-phosphorus metabolism, and regulated serum inorganic elements. Overall, female ESW exhibited a greater abundance of bioactive components and demonstrated superior anti-thrombotic and anti-osteoporotic effects. These findings highlight the superior therapeutic effects of female ESW due to its enriched bioactive components, supporting its clinical preference while underscoring the potential of male ESW for uilization of resource.

**Supplementary Information:**

The online version contains supplementary material available at 10.1038/s41598-025-90100-5.

## Introduction

*Eupolyphaga sinensis* Walker (ESW), also known as Tubiechong, Tuyuan, Dibiechong, Dibie and Zhechong in Chinese, belongs to the family Corydiidae *Blattodea* and is widely distributed in Southeast Asian countries, such as China, Thailand, India and Malaysia^1,2^. In China, ESW was first recorded as a drug in *Shen Nong Ben Cao Jin* (originating in the third century AD)^3^. The ESW have been artificially bred in the 1970s, and its breeding has become an industry. It can promote blood circulation and remove stasis, as well as facilitate the healing of tendons and bones. Clinical practice commonly involves the treatment of injuries, fractures, amenorrhea, postpartum stasis, tumors, and various other diseases^4^. When used as a medicine, ESW has various forms of application. The methods of administration consist of fresh application, dry grinding, and making decoctions, with oral and topical being the most frequently used^5–7^. The *Chinese Pharmacopoeia* (2020 edition) holds official status, including only the use of dried female ESW bodies for medicinal purposes^8^. This has resulted in a considerable waste of male ESW resources during the breeding process. However, why are female ESW designated for clinical application? To date, no reports have revealed their scientific implications.

Building on this historical context, recent analyses have revealed that ESW, as an insect-derived medicine, contains a variety of substances^4^. Small molecular compounds present in earthworms primarily include amino acids, fatty acids, alkaloids, volatile oils, and inorganic elements. Large molecular compounds include proteins, peptides, and polysaccharides. Previous research on the chemical composition of ESW was fragmented and superficial. These articles focused on female ESW and targeted large molecules such as proteins or polypeptides that cannot be directly absorbed by the human body^9,10^. This limitation restricts the understanding of other potentially significant components, such as small peptides, lipids, or low-molecular-weight compounds, which may play crucial roles in ESW’s therapeutic effects. In our preliminary study, we compared the primary metabolites present in female and male ESW and found that, in addition to the total protein content, female ESW exhibited significantly higher levels of total polysaccharides and crude fat than male ESW^11^. To our knowledge, there is currently no systematic study on the characterization of secondary metabolites in female and male ESW. Metabolomics techniques based on LC-MS have gained significant attention for analysing the chemical components of herbal medicines^12^. Their high-throughput fragmentation data were used for precise compound identification. By employing metabolomics, our study systematically investigates the bioactive small molecules in both male and female ESW, providing a comprehensive comparative analysis that addresses critical gaps in previous research.

Despite these advances in understanding the chemical composition of ESW, its broader pharmacological properties and clinical applications remain insufficiently explored. According to statistics, there are more than 200 Chinese patent medicines derived from the ESW^13^. ESW has been used in Chinese folk and traditional medical practices to treat various diseases including cardiovascular disease, orthopedic disease, and liver disease^14–16^, but there is still a lack of systematic experimental research, particularly in vivo pharmacological experiments. In recent years, pharmacological studies focused on the effect of promoting blood circulation and removing blood stasis of female ESW^17,18^, and ignored its effect of reinforcing tendon and bone, leading to a limited understanding of its overall therapeutic effects. Apart from a few early reports on the in vitro activities of female ESW^19^, there is a lack of comprehensive research that can accurately represent the efficacy of the ESW. Importantly, to our knowledge, there is currently no comparative study on the in vivo pharmacological effects between female and male ESW. Furthermore, the current edition of the *Chinese Pharmacopoeia* stipulates that only female ESW should be used in medicinal preparations, and there is a lack of standardized quality control components. This has resulted in inconsistent quality of the ESW medicinal materials and a significant waste of male ESW resources.

**Fig. 1:**
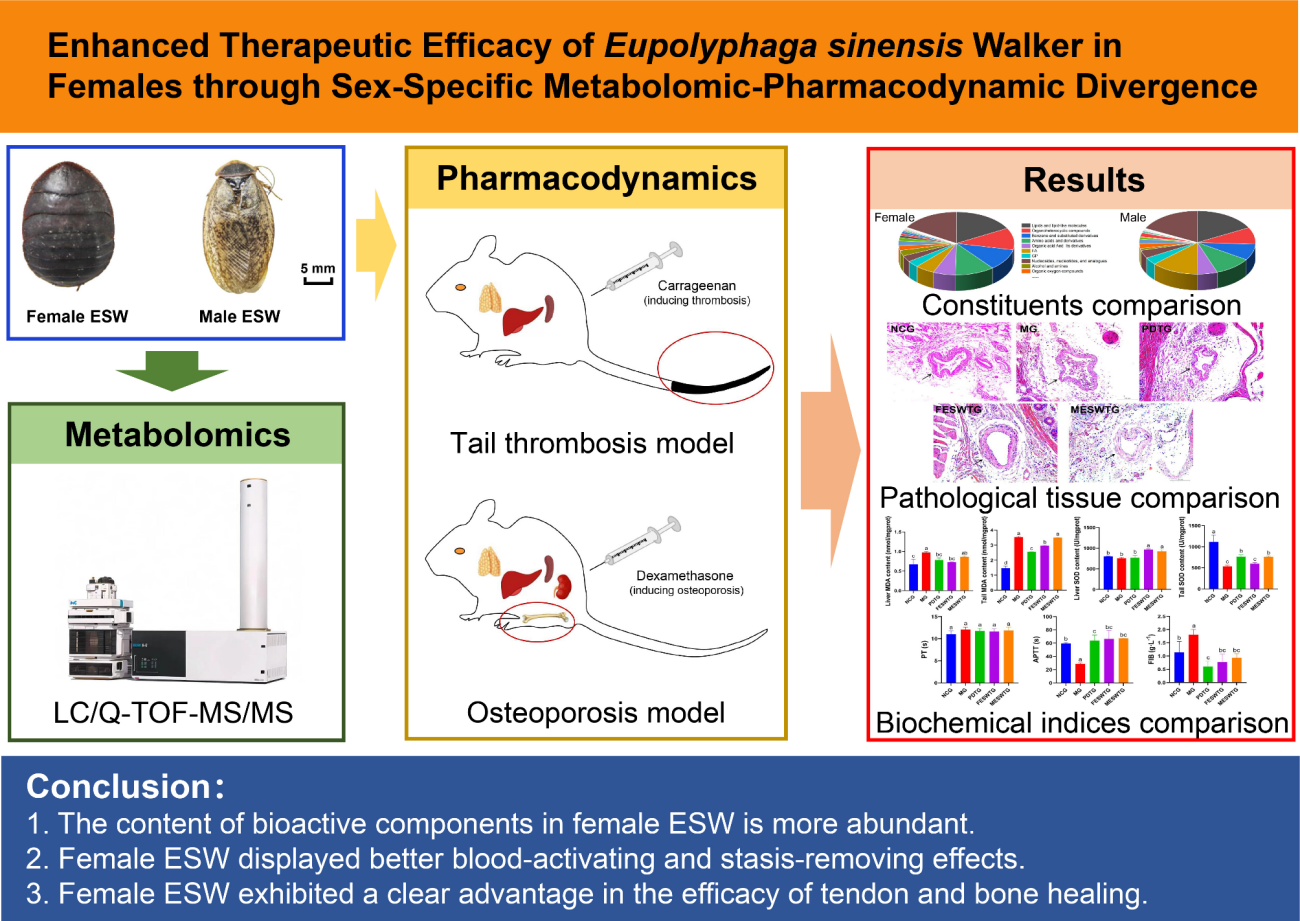
The flowchart of the research.

This study aims to investigate the compositional differences between female and male ESW using metabolomics and to evaluate their pharmacological effects in treating thrombosis and osteoporosis through in vivo experiments. This study world clarify the scientific significance of using female ESW in medicine and to provide a basis for the quality evaluation of ESW materials and the development and utilization of male ESW. The flowchart of the research is shown in Fig. 1.

## Results

### Differences in constituents between female and male ESW

#### Quality control results

As shown in Figure S1, the total ion chromatogram (TIC) curves of mass spectrometry detection of different quality control (QC) samples overlapped. The high degree of overlap indicates that the mass spectrometer has good signal stability when detecting the same sample at different times, ensuring good repeatability and reliability of the data.

#### The overall composition of female and male ESW

A total of 18,995 compound signals were detected in the positive electrospray ionization (ESI+) mode, of which 3595 had secondary identification information. A total of 17,238 substance signals were detected in the negative electrospray ionization (ESI-) mode, of which 816 had secondary identification information.

The components detected in male and female ESW can be divided into 31 categories. Thirty-one types were detected in the ESI + mode. The relative percentages of lipids and lipid molecules, amino acids and their derivatives in both female and male ESW exceeded 10%. The female ESW also contained organic heterocyclic compounds, benzene and substituted derivative. Twenty-five types were detected in ESI- mode, and the relative percentages of the fatty acids in both male and female ESW exceeded 20%. The relative contents of various components in female and male ESW are listed in Table S1. In addition, we also analysed the top 10 components expressed in male and female ESW. These components include phosphatidic acid (22: 0/a-15: 0), lysophosphatidylcholine (16: 0/0: 0), phenylalanine, hypoxanthine, uric acid and others, which were dominated by amino acids and fatty acids, suggesting that they may be the main pharmacoactive substances of ESW (Table 1).

**Table 1:** Top 10 highest components in female and male *Eupolyphaga sinensis* Walker.

No.	Compounds	Formula	Score	RT (min)	Mode	Group
1	PA (22: 0/a-15: 0)	C_40_H_79_O_8_P	0.98	11.18	ESI+	Female/male
2	L-Phenylalanine	C_9_H_11_NO_2_	0.86	1.81	ESI+	Female/male
3	Hypoxanthine	C_5_H_4_N_4_O	0.85	1.30	ESI+	Female/male
4	Carnitine C3: 0	C_10_H_19_NO_4_	0.81	1.23	ESI+	Female/male
5	LPC (16: 0/0: 0)	C_24_H_50_NO_7_P	0.81	8.83	ESI+	Female/male
6	Heptadecanoic acid	C_17_H_34_O_2_	0.96	9.19	ESI-	Female/male
7	Uric acid	C_5_H_4_N_4_O_3_	0.85	1.14	ESI-	Female/male
8	Choline	C_5_H_14_NO_+_	0.85	1.02	ESI+	Female
9	L-Tryptophan	C_11_H_12_N_2_O_2_	0.84	2.35	ESI+	Female
10	2- Hydroxyiso-caproic Acid	C_6_H_12_O_3_	0.82	3.48	ESI-	Female
11	Nicotinamide	C_6_H_6_N_2_O	0.83	1.39	ESI+	Male
12	Carnitine iso C4: 0	C_11_H_21_NO_4_	0.85	2.05	ESI+	Male
13	Inosine	C_10_H_12_N_4_O_5_	0.85	1.31	ESI-	Male

RT, retention time; VIP, variable importance in projection; ESI+, positive mode; ESI-, negative mode.

#### Multivariate statistical analysis

**Fig. 2:**
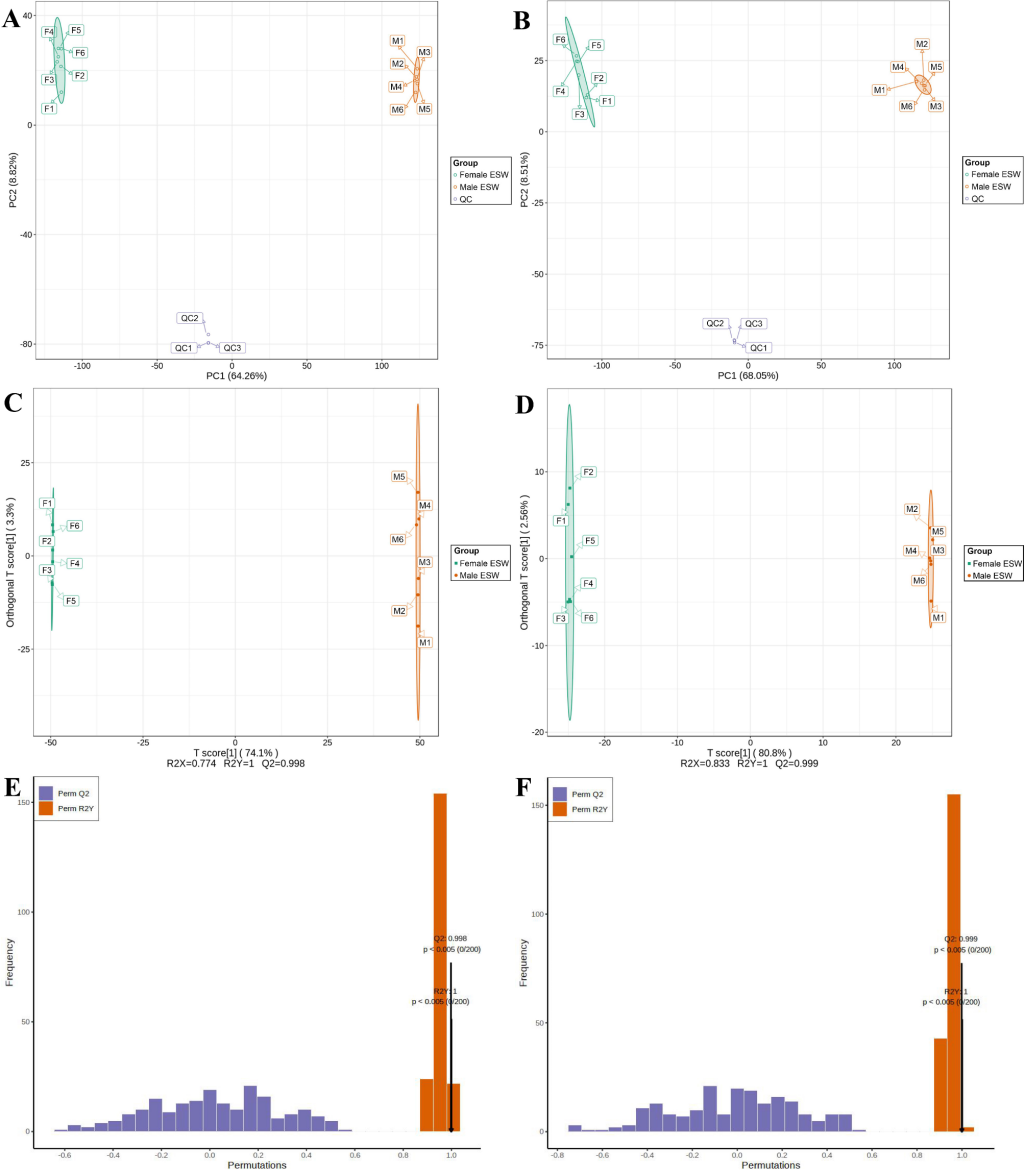
Multivariate statistical analysis plot based on the metabolome of the ESW. PCA score plot under (**A**) positive mode and (**B**) negative mode; PLS-DA score plot under (**C**) positive mode and (**D**) negative mode; validation results of the OPLS-DA model under (**E**) positive mode and (**F**) negative mode. PCA, principal component analysis; OPLS-DA, orthogonal partial least squares discriminant analysis; QC, quality control; ESW, *Eupolyphaga sinensis* Walker.

The principle component analysis (PCA) results are shown in Fig. 2A and B. The QC samples are gathered, indicating that there is no obvious deviation in each step of the experiment. The separation trend of each group in the direction of PC1 is obvious, indicating that the difference between groups is significant.

The orthogonal partial least squares discriminant analysis (OPLS-DA) model was established based on metabolites in female and male ESW. The score plot is shown in Fig. 2C and D, which further demonstrates the differences between male and female ESW. R^2^X and R^2^Y represent the model interpretation rates of the X and Y matrices, respectively. The closer the two indices are to 1, the more stable and reliable the model is. Q^2^ represents the predictive ability of the model. Q^2^ > 0.5 can be regarded as an effective model, and Q^2^ > 0.9 is considered an excellent model. The results of the model constructed in this study are as follows: ESI+, R^2^X = 0.774, R^2^Y = 1, Q^2^ = 0.998; ESI-, R^2^X = 0.833, R^2^Y = 1, Q^2^ = 0.999. The OPLS-DA model is stable, reliable and excellent. The model verification results are shown in Fig. 2E and F. The abscissa indicates the accuracy of the model, and the ordinate indicates the frequency of the classification effect of the model. *p*<0.05 indicated that the model did not overfit. Among the 200 random verifications of the models constructed in this study, 0 models had better prediction capabilities and explanation rates for the Y matrix than this model. *p*<0.005 indicated that the model constructed in this experiment is reliable.

#### Analysis of differential components in the female and male ESW

**Fig. 3:**
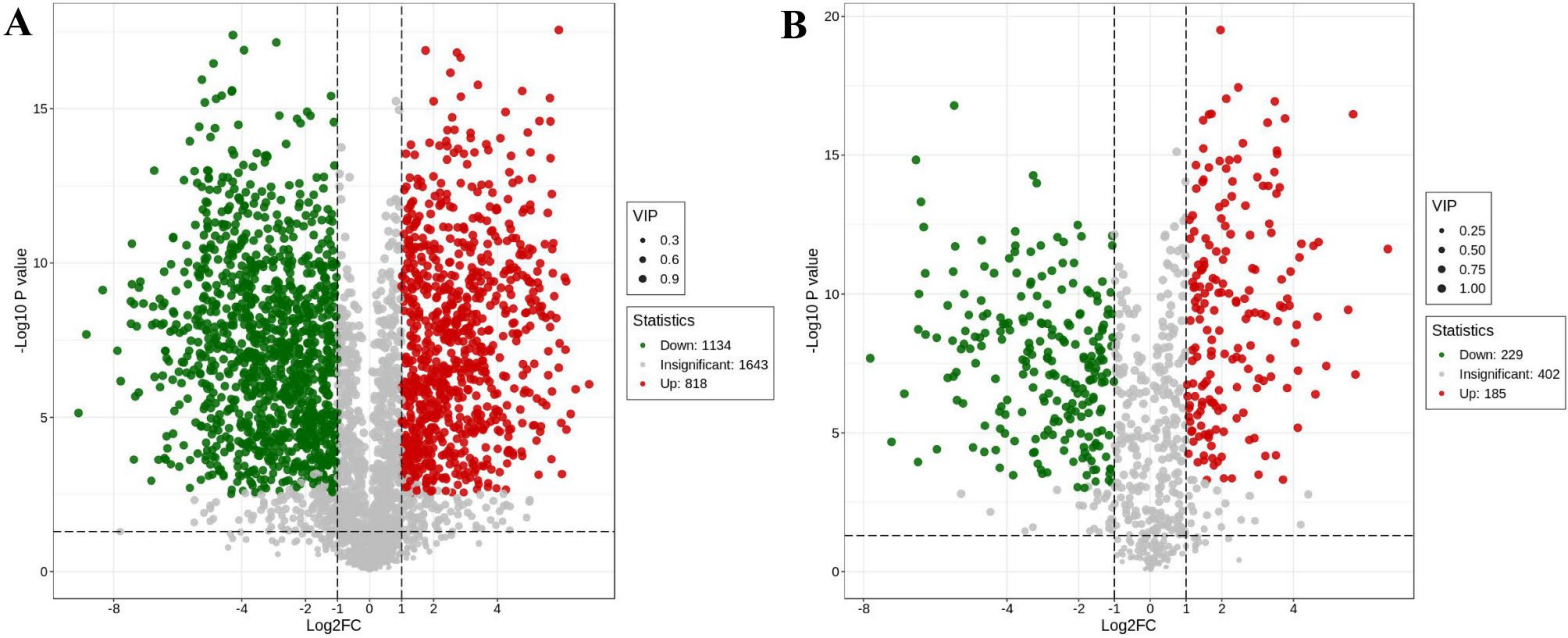
Volcano plot of differential components between female and male ESW. (**A**) Positive mode; (**B**) Negative mode. ESW, *Eupolyphaga sinensis* Walker.

In the ESI + and ESI- modes, 1952 and 414 differential components were screened out respectively. The difference is shown in Fig. 3. The abscissa represents the FC value (taken as the logarithm with base 2). The ordinate represents the P value (taken as the logarithm of base 10). The size of the scatter points indicates the variable importance in projection (VIP) value. Red represents components that are significantly up-regulated in male ESW compared to female ESW, green represents components that are significantly down-regulated in expression, and gray represents components with no significant difference. The statistical results of the number of up- and down-regulated differential components are shown in Table S2. The types and quantities of differential components are shown in Table S3. The differential components can be divided into 30 types.

#### Bioinformatics analysis of differential components

**Fig. 4:**
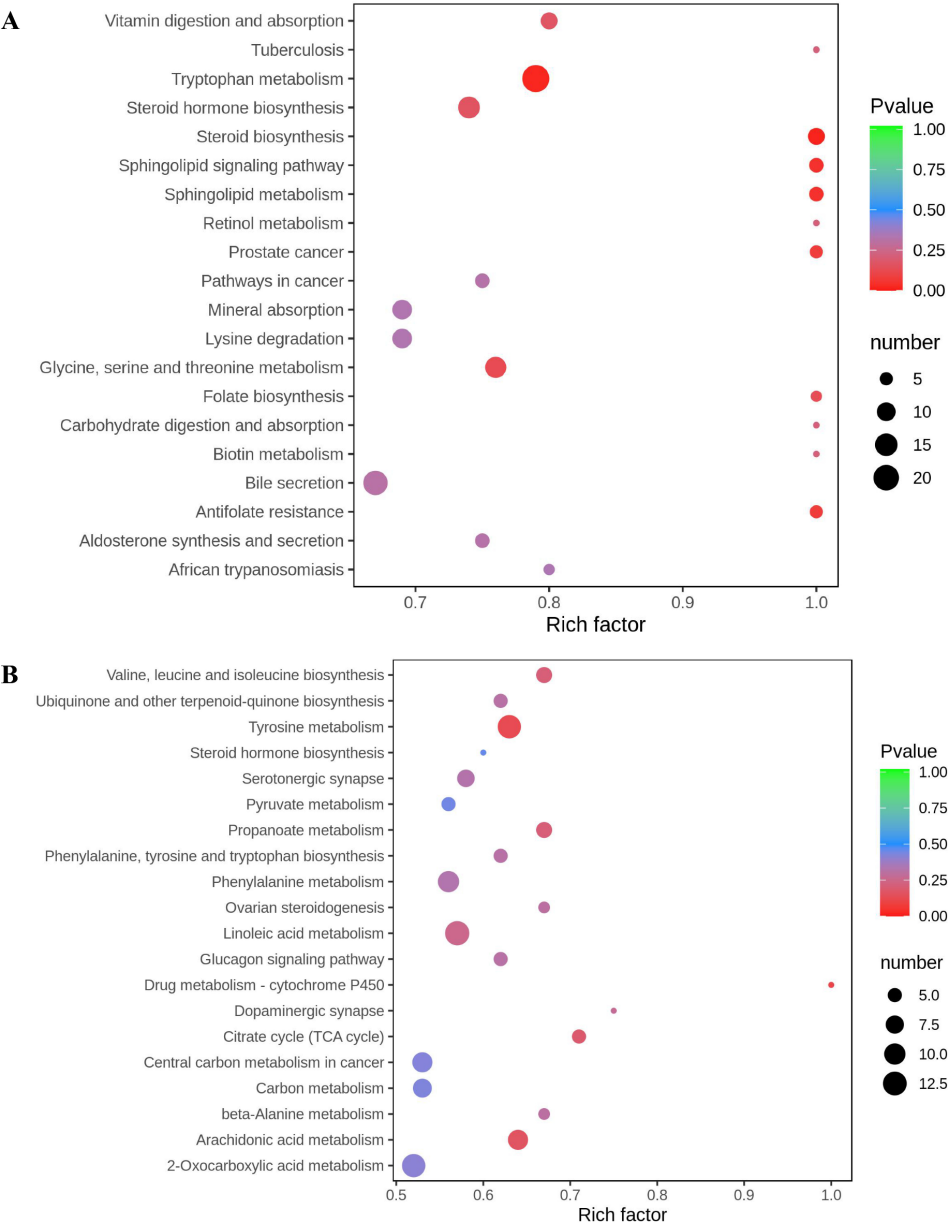
Bubble plot of KEGG enrichment analysis for differential components between female and male ESW. (**A**) Positive mode; (**B**) Negative mode. KEGG, kyoto encyclopedia of genes and genomes; ESW, *Eupolyphaga sinensis* Walker.

In the ESI + mode, 337 differential components were annotated, which were enriched in 169 signalling pathways such as tryptophan metabolism and bile secretion. In the ESI- mode, 136 differential components were annotated, which were enriched in 104 signalling pathways, such as linoleic acid and arachidonic acid metabolism. The top 20 pathways ranked by enrichment are shown in Fig. 4. The rich factor represents the ratio of the number of differential components in the corresponding pathway to the total number of components annotated by the pathway. The size of the point reflects the number of differential components enriched in the corresponding pathway. The color of the point reflects the *p* value, and the redder the point is, the more significant the enrichment.

#### Analysis of advantageous components in the female ESW

There are many different components between male and female ESW, but few are known to have pharmacological activity. Compared with those in male ESW, 8 pharmacologically active compounds, 15 small peptides, and 13 prostaglandins were up-regulated, while 8 small peptides and 9 prostaglandins were down-regulated in female ESW. More details are listed in Table 2. In general, the content of pharmacologically active components in female ESW was higher than that in male ESW.

**Table 2:** Advantageous components of female *Eupolyphaga sinensis* Walker vs. males.

No.	Types	Compounds	Formula	Score	RT (min)	VIP	Trend	Mode
1	Active compound	Adenosine	C_10_H_13_N_5_O_4_	0.84	1.69	1.16	up	ESI+
2		3-Hydroxy-2-methyl-4 H-pyran-4-one	C_6_H_6_O_3_	0.82	2.59	1.16	up	ESI+
3		Penbutolol	C_18_H_29_NO_2_	0.73	5.37	1.16	up	ESI+
4		L-Tryptophan	C_11_H_12_N_2_O_2_	0.85	2.34	1.11	up	ESI-
5		Phenyllactate(Pla)	C_9_H_10_O_3_	0.84	3.83	1.11	up	ESI-
6		Dihydroferulic Acid	C_10_H_12_O_4_	0.81	3.88	1.11	up	ESI-
7		Protocatechuic Acid	C_7_H_6_O_4_	0.85	2.41	1.11	up	ESI-
8		Chenodeoxycholic Acid	C_24_H_40_O_4_	0.83	8.20	1.11	up	ESI-
9	Small peptide	Pro-Ile	C_11_H_20_N_2_O_3_	0.71	3.61	1.16	up	ESI+
10		Leu-Val	C_11_H_22_N_2_O_3_	0.68	2.09	1.16	up	ESI+
11		Ala-Phe	C_12_H_16_N_2_O_3_	0.71	2.22	1.13	up	ESI+
12		Leu-Ala	C_9_H_18_N_2_O_3_	0.65	2.21	1.16	up	ESI+
13		Glu-Tyr	C_14_H_18_N_2_O_6_	0.74	1.84	1.14	down	ESI+
14		Thr-Phe	C_13_H_18_N_2_O_4_	0.69	1.88	1.10	up	ESI+
15		Leu-Leu-Phe	C_21_H_33_N_3_O_4_	0.56	3.26	1.15	up	ESI+
16		Val-Val-Val	C_15_H_29_N_3_O_4_	0.61	3.17	1.04	up	ESI+
17		Glu-Leu	C_11_H_20_N_2_O_5_	0.80	2.25	1.16	up	ESI+
18		Leu-Ala-Val	C_14_H_27_N_3_O_4_	0.75	2.06	1.16	up	ESI+
19		Tyr-Phe	C_18_H_20_N_2_O_4_	0.76	3.96	1.16	up	ESI+
20		Gly-Phe-Phe	C_20_H_23_N_3_O_4_	0.53	4.91	1.16	up	ESI+
21		Glyc-Pro	C_7_H_12_N_2_O_3_	0.52	1.62	1.16	down	ESI+
22		Leu-Leu-Gly	C_14_H_27_N_3_O_4_	0.58	1.69	1.14	up	ESI+
23		Ile-Ile-Thr	C_16_H_31_N_3_O_5_	0.61	3.04	1.02	up	ESI+
24		Pro-His	C_11_H_16_N_4_O_3_	0.68	1.32	1.15	up	ESI+
25		Leu-Phe	C_15_H_22_N_2_O_3_	0.57	3.42	1.16	down	ESI+
26		Anserine	C_10_H_16_N_4_O_3_	0.54	5.08	1.11	up	ESI+
27		Phe-Phe	C_18_H_20_N_2_O_3_	0.73	3.25	1.10	down	ESI+
28		Leu-Gly	C_8_H_16_N_2_O_3_	0.59	4.18	1.15	down	ESI+
29		Ala-Tyr	C_21_H_25_N_3_O_6_	0.81	1.71	1.15	down	ESI+
30		Phe-Ala	C_12_H_16_N_2_O_3_	0.97	2.27	1.03	down	ESI+
31		Tyr-Leu	C_15_H_22_N_2_O_4_	0.67	1.25	1.11	down	ESI-
32	Prostaglandin	13,14-dihydro-15-keto Prostaglandin D1	C_20_H_34_O_5_	0.62	6.23	1.16	up	ESI+
33		8-iso-15-Ketoprostaglandin E2	C_20_H_30_O_5_	0.75	7.20	1.11	up	ESI+
34		15-keto Latanoprost(free acid)	C_23_H_32_O_5_	0.73	6.23	1.16	up	ESI+
35		Prostaglandin F3α	C_20_H_32_O_5_	0.73	11.71	1.09	up	ESI-
36		Prostaglandin E2-1-glyceryl ester	C_23_H_38_O_7_	0.57	5.53	1.16	down	ESI+
37		6-ketoProstaglandin E1	C_20_H_32_O_6_	0.87	5.68	1.16	up	ESI+
38		Dinorprostaglandin E1	C_18_H_30_O_5_	0.68	7.03	1.11	down	ESI-
39		Prostaglandin F2β	C_20_H_34_O_5_	0.59	6.23	1.16	up	ESI+
40		Prostaglandin F2 Ethanolamide-d4	C_22_H_35_D_4_NO_5_	0.50	9.67	1.16	up	ESI+
41		Prostaglandin A1 ethyl ester	C_22_H_36_O_4_	0.70	9.86	1.10	up	ESI+
42		Prostaglandin F1α	C_20_H_36_O_5_	0.64	6.42	1.15	up	ESI+
43		12-Demethyl-11,12-dehydropaspaline	C_27_H_35_NO_2_	0.51	9.67	1.16	up	ESI+
44		Prostaglandin H1	C_20_H_34_O_5_	0.73	7.70	1.09	up	ESI+
45		11-Deoxyprostaglandin E2	C_20_H_32_O_4_	0.57	6.54	1.07	down	ESI+
46		Prostaglandin F1a-d9	C_20_H_27_D_9_O_5_	0.54	6.90	1.16	up	ESI+
47		2,3-Dinor-8-epi-prostaglandin F2alpha	C_18_H_30_O_5_	0.57	7.03	1.09	down	ESI+
48		Prostaglandin D2 1-glyceryl ester	C_23_H_38_O_7_	0.65	6.30	1.02	down	ESI+
49		5-trans-Prostaglandin F2α	C_20_H_34_O_5_	0.58	4.42	1.14	up	ESI+
50		Prostaglandin I2	C_20_H_32_O_5_	0.69	5.95	1.10	down	ESI+
51		17-Phenyltrinorprostaglandin D2	C_23_H_30_O_5_	0.67	1.73	1.15	down	ESI+
52		Prostaglandin G2	C_20_H_32_O_6_	1.00	5.73	1.16	down	ESI+
53		15(S)-15-Methylprostaglandin E2	C_21_H_34_O_5_	0.57	4.54	1.16	down	ESI+

RT, retention time; VIP, variable importance in projection; ESI+, positive mode; ESI-, negative mode.

### Differences of anti-thrombotic effect between female and male ESW

#### Impact of female and male ESW on the tail thrombosis rate

**Fig. 5:**
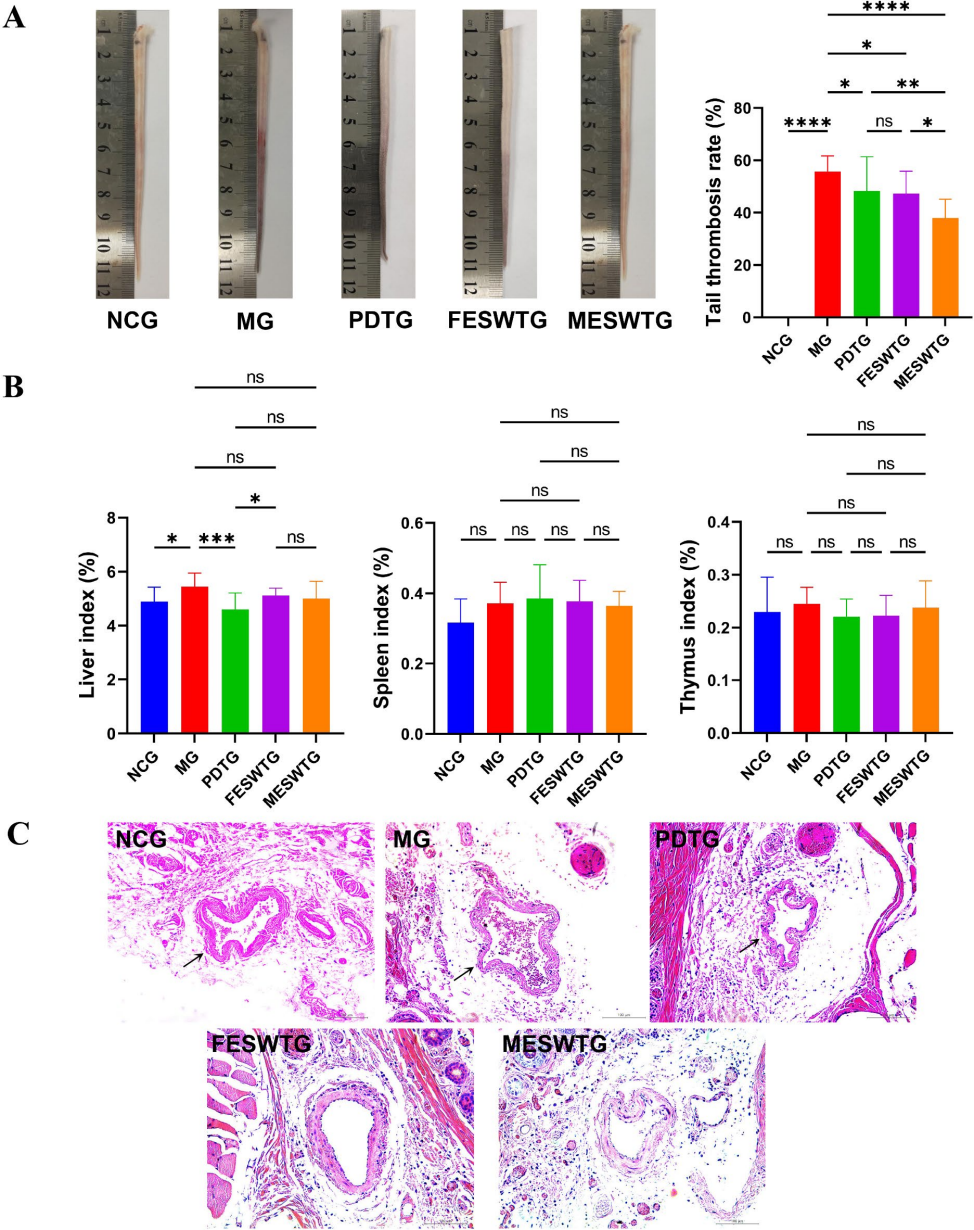
Differences of anti-thrombotic effect between female and male ESW. (**A**) Mouse tail thrombosis status and rate; (**B**) Organ indices of liver, spleen and thymus; (**C**) Hematoxylin and eosin staining of tail tissue. Nucleus was stained purple-blue and cytoplasm was stained red. The black arrows indicate the accumulation of blood cells in the tail vein of the mice and the condition of the blood vessels. The MG showed significant accumulation of blood cells in the tail vessels, while the accumulation was reduced in the treatment groups. The tail vessels in both FESWGT and MESWGT were more dilated compared to the MG. The effect of female ESW was better than males. The data are expressed as mean ± SD (*n* = 10). Significant differences are indicated as follows: ∗*p* < 0.05, ∗∗*p* < 0.01, ∗∗∗*p* < 0.001, ∗∗∗∗*p* < 0.0001; ns, not significant. NCG, normal control group; MG, model group; PDTG, positive drug treatment group; FESWTG, female ESW treatment group; MESWTG, male ESW treatment group; ESW, *Eupolyphaga sinensis* Walker.

The status of the mouse tail thrombus in each group is shown in Fig. 5A. The black tail of the mice in the model group (MG) was the most severe, and the symptoms were relieved after administration. The female ESW treatment group (FESWTG) had the mildest degree. The tail thrombosis rate in the MG was significantly higher than that in the NCG (*p*<0.0001) (Fig. 5A). Compared with those in the MG, the values in each treatment group decreased significantly. The tail thrombosis rate in the male ESW treatment group (MESWTG) was significantly lower than that in the FESWTG (*p*<0.05), and the latter was equivalent to the treatment effect of positive drug.

#### Effects of female and male ESW on organ indices

The organ indices are shown in Fig. 5B. Compared with that of the normal control group (NCG), the liver index of the MG increased significantly (*p*<0.05), indicating liver damage. The liver index of treatment groups showed a downward trend, but only the positive drug treatment group (PDTG) had a significant difference (*p*<0.001). For the spleen and thymus indices, there was no significant difference among groups.

#### Effects of female and male ESW on tail tissue histopathology

The pathological cross-section of the mouse tail vein is shown in Fig. 5C, which reflects the overall state of the tail vein blood vessels (black arrows) of mice in each group and the accumulation of blood cells in the blood vessels. Compared with those in the NCG, the accumulation of blood cells in the mouse tail vein in the MG significantly increased, and the blood vessels showed contraction. Compared with that in the MG, the number of blood cells in the mouse tail vein in all treatment groups significantly decreased, and the blood vessel shrinkage was alleviated. The therapeutic effect of the FESWTG was better than that of the NESWTG and PDTG.

#### Effects of female and male ESW on MDA and SOD activity in liver and tail tissues

**Fig. 6:**
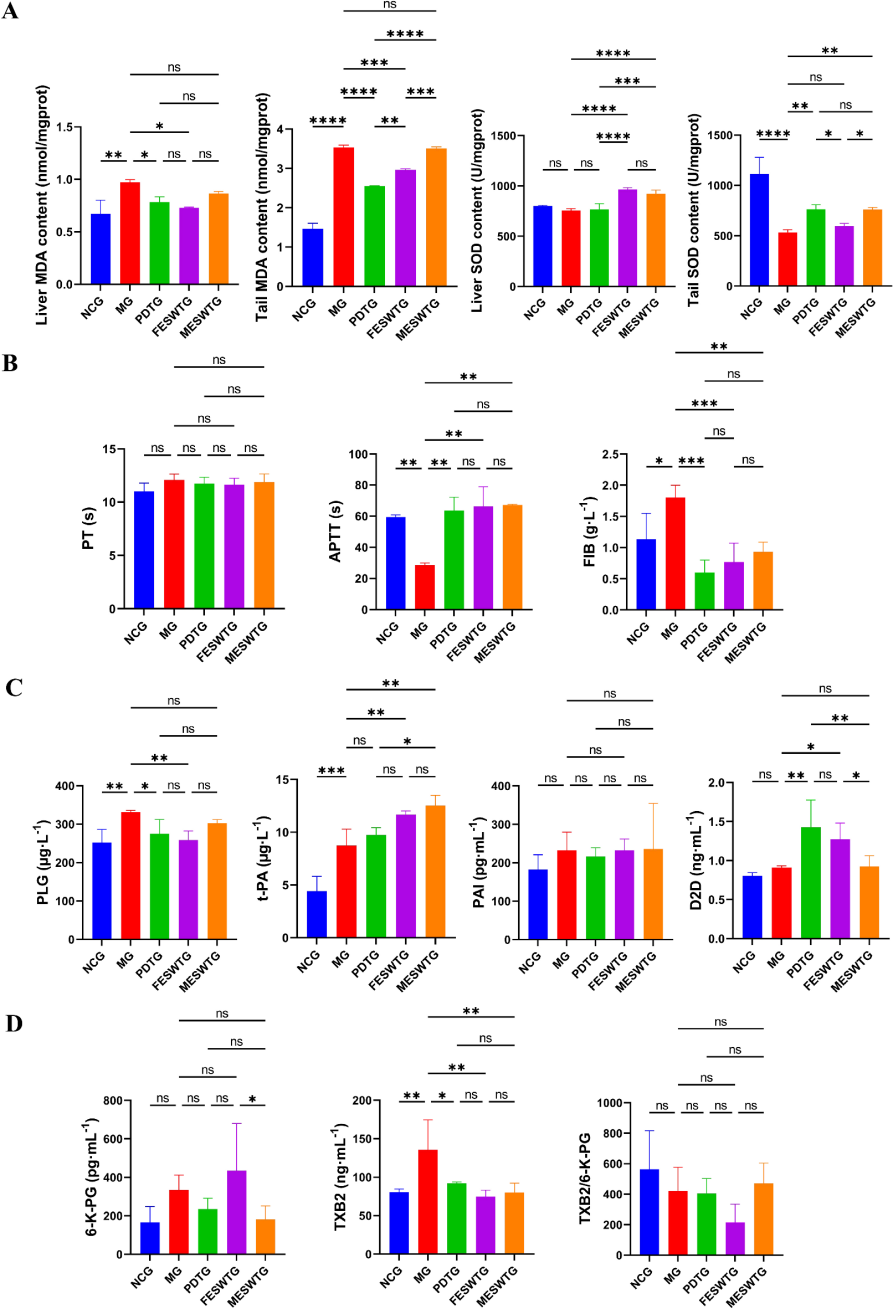
Effects of female and male ESW on plasma biochemical indicators in osteoporotic rats. (**A**) MDA content in the liver and tail; (**B**) Coagulation indicators; (**C**) Fibrinolysis indicators; (**D**) Vascular contraction and relaxation, platelet function indicators. The data are expressed as mean ± SD (*n* = 10). Significant differences are indicated as follows: ∗*p* < 0.05, ∗∗*p* < 0.01, ∗∗∗*p* < 0.001, ∗∗∗∗*p* < 0.0001; ns, not significant. NCG, normal control group; MG, model group; PDTG, positive drug treatment group; FESWTG, female ESW treatment group; MESWTG, male ESW treatment group; MDA, malondialdehyde; SOD, superoxide dismutase; PT, prothrombin time; APTT, activated partial prothrombin time; FIB, fibrinogen; PLG, plasminogen; t-PA, tissue-type plasminogen activator; PAI, plasminogen activator inhibitor; D2D, d-dimer; 6-K-PG, 6-keto‐prostaglandin; TXB2, thromboxane b2; ESW, *Eupolyphaga sinensis* Walker.

Malondialdehyde (MDA) and superoxide dismutase (SOD) in tissue can reflect the degree of cell damage and antioxidant capacity, respectively. Combining these two indicators can reflect organizations’ coping strategies for oxidative stress (Fig. 6A). The liver and tail MDA content in the MG was significantly higher than that in the NCG. Treatment with ESW resulted in a decrease in the above two indicators, and the FESWTG showed a significant effect (*p*<0.05 or 0.001). The tail SOD content in the MG was significantly lower than that in the NCG and it significantly increased after administration in the PDTG and MESWTG (*p*<0.01). In addition, the liver SOD content in the FESWTG and MESWTG was significantly higher than that in the MG (*p*<0.05), and there was no significant difference between the two groups.

#### Effects of female and male ESW on blood plasma biochemical indices

Indices related to coagulation system activity include plasma prothrombin time (PT), activated partial prothrombin time (APTT) and fibrinogen (FIB). Plasma plasminogen (PLG), tissue-type plasminogen activator (t-PA), plasminogen activator inhibitor (PAI), and d-dimer (D2D) are related to fibrinolytic system activity. Moreover, 6-keto-prostaglandin (6-K-PG) and thromboxane b2 (TBX2) are related to vasomotor function and platelet activity, respectively. The results of these indicators are shown in Fig. 6B-D. Compared with NCG, MG showed significant increase in plasma levels of FIB, PLG, t-PA, and TXB2. After administration, the above indicators decreased to varying degrees, while t-PA in the FESWTG and MESWTG significantly increased. Furthermore, the APTT level of the MG was significantly lower than that of the NCG (*p*<0.01) and it significantly increased after administration. Notably, the D2D and 6-K-PG contents in the FESWTG were significantly higher than those in the MESWTG (*p*<0.05). There was no significant difference in PT or PAI among groups. Overall, female and male ESW have different degrees of improvement in thrombosis-related indicators, but the regulation of MDA, PLG, D2D, TXB2/6-K-PG levels was more significant in female ESW group, indicating the effect of the female ESW was better than that of male ESW.

### Differences of anti-osteoporotic effect between female and male ESW

#### Effects of female and male ESW on femoral biomechanical parameters

**Fig. 7:**
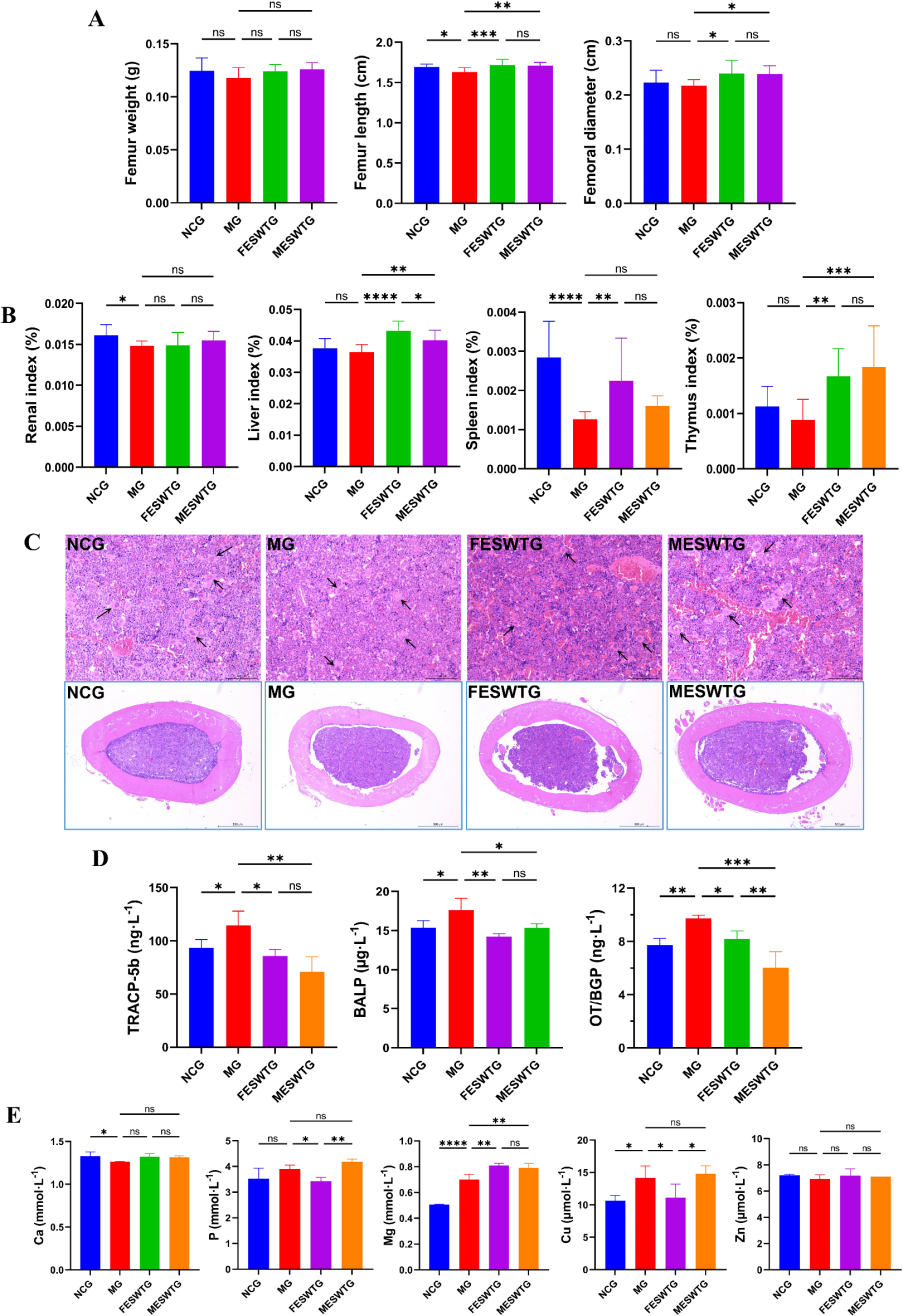
The differences of anti-osteoporotic effect between female and male ESW. (**A**) Femoral biomechanical parameters (*n* = 10); (**B**) Organ index of renal, liver, spleen and thymus (*n* = 10); (**C**) Hematoxylin and eosin staining of right femur. Nucleus was stained purple-blue and cytoplasm was stained red. The black arrows indicate the trabeculae. In the NCG, the trabeculae were evenly distributed, with larger individual trabecular areas and intact bone cavity structures. In the MG and treatment groups, the number and area of trabeculae were reduced, and there were gaps in the bone cavities. The number of trabeculae in both the FESWGT and MESWGT was higher than in the MG. (**D**) TRACP-5b, BALP and OT/BGP levels serum (*n* = 3); (**E**) Content of inorganic elements in serum (*n* = 3). Data were expressed as mean ± SD. The significance of difference is indicated as ∗*p* < 0.05, ∗∗*p* < 0.01, ∗∗∗*p* < 0.001, ∗∗∗∗*p* < 0.0001; ns, not significant. NCG, normal control group; MG, model group; FESWTG, female ESW treatment group; MESWTG, male ESW treatment group; TRACP-5b, tartrate-resistant acid phosphatase 5b; BALP, bone alkaline phosphatase; OT/BGP, osteocalcin/bone glutamate protein; ESW, *Eupolyphaga sinensis* Walker.

The weight, length and diameter of the mice right femur are shown in Fig. 7A. The femur length and diameter of the MG were lower than those of the NCG. Both female and male ESW significantly increased the above indicators. There was no significant difference in the femoral weight of mice in each group.

#### Effects of female and male ESW on organ indices

The organ indices are shown in Fig. 7B. Compared with those of the NCG, the renal, liver, spleen and thymus indices of the MG reduced to variable degrees. After treatment with ESW, other indices increased significantly, except for the renal index. The therapeutic effect of female ESW was significantly better than that of male ESW.

#### Effects of female and male ESW on femoral tissue histopathology

Pathological cross-sections of the mice right femur are shown in Fig. 7C. The bone cavity structure of mice femur in the NCG is complete, the bone trabeculae (black arrows) are evenly distributed, and the area of a single bone trabecula is large. There are gaps in the bone cavities of the MG, FESWTG and MESWTG. Compared with those in the NCG, the number and area of trabecular bone in the MG were less. However, the number of trabeculae in the FESWTG and MESWTG was more than that in the MG.

#### Effects of female and male ESW on blood serum biochemical indices

The serum tartrate-resistant acid phosphatase 5b (TRACP-5b), bone alkaline phosphatase (BALP) and osteocalcin/bone glutamate protein (OT/BGP) levels related to bone metabolism are shown in Fig. 7D. The above three indicators in the MG were significantly higher than that in the NCG. Both female and male ESW significantly reduced these indicators.

The serum Ca, P, Mg, Cu, and Zn levels are shown in Fig. 7E. Compared with those in the NCG, the serum Ca levels in the MG were significantly reduced (*p*<0.05), and the P, Mg and Cu levels were significantly increased (*p*<0.05). After administration, there was a callback in the serum Ca, P and Cu levels except for Mg. The improvement effect of female ESW on P and Cu is better than that of male ESW. There was no significant difference in serum Zn level. Overall, female and male ESW have different degrees of improvement in osteoporosis-related indicators, but the TRACP-5b, OT/BGP, P and Cu levels of female ESW group were closer to the normal control group, indicating that the effect of female ESW was better than that of male ESW.

## Discussion

This study employed ultra performance liquid chromatography-quadrupole-time of flight-mass spectrometry (UPLC-Q-TOF-MS/MS) technology to detect approximately 31 types of components in female and male ESW. The analysis revealed that the relative percentages of lipids and lipid molecules, amino acids and their derivatives, and fatty acids exceeded 10%. In addition to the compounds mentioned above, the percentages of organic heterocyclic compounds, benzene, and its derivatives in female ESW is also found to exceed 10%. However, these components only account for approximately 8% in male ESW. Among the small molecule compounds found in ESW, lipids are the most abundant and largest class, which aligns with previous literature^13^. Considering the low polarity of these compounds, it is recommended to use solvents with low polarity, such as ethanol, for extraction in clinical applications. Seven of the top ten ingredients in male and female land turtles are the same, including phosphatidic acid (22:0/a-15:0) and lysophosphatidylcholine (16: 0/0: 0) (involved in the composition of various biofilms), phenylalanine (produced by protein metabolism), hypoxanthine (produced by nucleic acid metabolism), uric acid (produced by purine metabolism), unsaturated fatty acid heptadecanoic acid (produced by fat metabolism), and amino acid alanine acylcarnitine (promotes fat metabolism). The unique ingredients in female ESW include tryptophan, choline and 2-hydroxy-4-methylpentanoic acid. Choline is related to glucose and lipid metabolism and can prevent the deposition of cholesterol in blood vessels^20^. Niacinamide, isobutyl-L-carnitine and inosine are unique compounds in male ESW. Nicotinamide is a B3 vitamin, related to sugar and protein metabolism, and has a recognized anti-aging effect on skin^21^. Inosine is an auxiliary hepatoprotective drug. However, no studies have reported on the metabolome of the ESW. Furthermore, the components that differ significantly between female and male ESW are primarily lipids that are prone to producing aflatoxins during the storage of medicinal materials^22^, suggesting that low-temperature storage should be employed, and the storage time should be minimized.

The ingredients contained in male and female ESW are similar in number but differ in content. Compared with male ESW, the eight highly expressed active ingredients in female ESW include adenosine, (+)-cyclopentanol, L-tryptophan, DL-3-phenyl-2-hydroxypropionic acid, hydrogenated ferulic acid, protocatechuic acid, L-threonine and chenodeoxycholic acid. Modern pharmacological research shows that adenosine can regulate the cardiovascular system. As an essential amino acid, L-tryptophan has a wide range of physiological activities, including antioxidant, immunity improvement, and antidepressant activities^23^. DL-3-phenyl-2-hydroxypropionic acid has broad-spectrum antibacterial activity. Ferulic acid has been proven to have anti-thrombotic, analgesic, and promotion of osteoblast proliferation and calcification effects^24,25^. Some studies have found that hydrogenated ferulic acid produced by gastrointestinal metabolism have stronger antiplatelet aggregation activity in vitro than its phenolic precursor^26^. Protocatechuic acid can also prevent platelet aggregation, is a new antithrombotic drug^27^, and can also regulate bone metabolism^28^. Moreover, chenodeoxycholic acid can dissolve and inhibit cholesterol absorption^29^. In addition, 15 small peptides and 13 prostaglandins were highly expressed in female ESW. Proteins and peptides are widely considered to be active macromolecular components of animal drugs^30^, but there is a lack of systematic research. Prostaglandins have a clear regulatory effect on vasomotion. The female ESW recorded in the *Chinese Pharmacopoeia* are used as medicines. These differential markers may be the material basis for why the female ESW is better than the male ESW in medicine. Further investigations, such as validating the in vitro activity of individual compounds, are warranted, along with exploring the mechanisms behind the observed differences in bioactive compounds and conducting clinical trials to validate the findings in human subjects.

The mouse tail thrombosis model is a widely used “blood stasis syndrome” model that has been included in authoritative monographs and is relatively simple to operate^31,32^. The inflammatory factors released by carrageenan-induced tail inflammation in mice cause damage to vascular endothelial cells and eventually induce thrombosis^33^. Aspirin enteric-coated tablets, a recognized antithrombotic drug, are often used as positive controls. The construction of the model is consistent with our research purpose. According to the status of the tail thrombus, the tail thrombosis rate and HE pathological sections, the model was judged to be successful.

Both male and female ESW can relax blood vessels and reduce blood cell accumulation. The female ESW had a better effect on reducing the degree of tail thrombosis, while the male ESW had a better effect on suppressing the tail thrombosis rate. The health of the liver is closely related to systemic blood circulation, while the health of the spleen and thymus is related to the body’s immune function and can reflect drug toxicity. By comparing the organ indices, we found that both male and female ESW have hepatoprotective effects, and the female ESW can also enhance immunity by increasing the spleen index. Notably, the immune-boosting activity of ESW makes it a candidate drug for the treatment of liver cancer^34^.

The formation of mouse tail thrombus is caused by inflammation, which often occurs together with oxidative stress, and the two processes promote each other. The anti-inflammatory and antioxidant effects of drugs can be evaluated by detecting oxidative stress indicators. Generally, higher MDA levels indicate more severe cell damage, and higher SOD activity indicates stronger antioxidant (anti-cell damage) capabilities. The MDA levels in the liver and tail tissues of the FESWTG were significantly lower than those in the liver and tail tissues of the MG, indicating that female ESW can protect cells from damage and effectively block the “oxidative stress-inflammation-malignant thrombosis” interaction network from the source^35^, while male ESW has no such effect. Interestingly, female ESW has also been reported to improve the antioxidant capacity of fish^36^.

The conversion of FIB into fibrin under thrombin action is the main mechanism of thrombosis^37^. The generation of thromboplastin involves two major pathways: endogenous and exogenous pathways. The initial coagulation factors activated are intravascular and extravascular. Due to different detection principles, PT reflects the activity of the exogenous coagulation system, while APTT reflects the activity of the endogenous coagulation system^38^. The higher the FIB content and the shorter the PT and APTT are, the stronger the activity of the coagulation system. There was no significant difference in PT between groups, indicating that neither modelling nor drug administration had any effect on the activity of the exogenous coagulation system, while both male and female ESW could weaken the activity of the endogenous coagulation system by reducing FIB content and prolonging APTT to exert anticoagulant effects. Notably, because we conducted in vivo efficacy studies, the results are more reliable than those of in vitro experiments^39^ and represent new findings.

The promotion of fibrinolysis by plasmin is the main mechanism by which it promotes fibrinolysis^40^. Plasmin is converted from PLG by activating factors such as t-PA. PAI inhibits the activation of the PLG by t-PA. D2D is a specific product of plasmin after dissolving fibrin^41^. The above indicators can reflect the activity of the body’s fibrinolytic system. The t-PA of both the FESWTG and MESWTG was significantly higher than that of the MG, but there was no significant difference in the PAI between the groups, indicating that both male and female ESW can enhance the activity of the fibrinolytic system by increasing the t-PA content. The difference is that the PLG content of the FESWTG was significantly lower than that of the MG, and the D2D content was significantly higher than that of the MG, indicating that the female ESW can also enhance the activity of the fibrinolytic system by reducing the PLG content. In summary, the profibrinolytic effect of female ESW is stronger than that of male ESW. These findings are similar to the previous literature^19^, but the indicators we detected are more comprehensive. Furthermore, we compared the effects of male and female ESW, and the results were obviously more convincing.

TXA2 promotes platelet aggregation and vasoconstriction, whereas PGI2 counteracts TXA2’s actions, exhibiting vasodilatory effects. The above two indices are dynamically balanced to maintain the normal diastolic function of blood vessels and platelet activity^42^. Since TXA2 and PGI2 are unstable, TXB2 and 6-K-PG are their stable end products. The lower the TXB2 content was, the lower the platelet activity. The smaller the TXB2/6-K-PG was, the more the blood vessels relaxed. The TXB2 content in both the FESWTG and MESWTG was significantly lower than that in the MG. Combined with the observation of pathological sections, both male and female ESW can relax blood vessels and resist platelet aggregation. The TXB2/6-K-PG ratio of the FESWTG was significantly lower than that of the NCG, but there was no significant difference between the MESWTG and the NCG, indicating that the female ESW has a better vasodilatory effect. Based on the above experimental results, it was proved that although both male and female ESW have the effect of promoting blood circulation and removing blood stasis, the efficacy of the female ESW is better than that of the male ESW.

The glucocorticoid-induced osteoporosis (GIOP) mouse is an important model for modern osteoporosis disease research. It has the advantages of being economical, easy to establish, and easy to operate. Models can be established by sustained-release, oral administration, and injection, among which intramuscular injection is the most controllable^43^. However, its pathophysiology differs from other models such as ovariectomy-induced and age-related models, which better mimic estrogen deficiency or gradual aging-related bone loss^44^. Each model has distinct strengths and weaknesses, with GIOP being ideal for exploring drug-induced osteoporosis mechanisms, while the ovariectomy and age-related models better replicate common clinical scenarios.

In our experiment, the femoral bone cavity of the mice in the MG was enlarged, the bone structure was destroyed, and the length of the femur was significantly shortened, indicating that the model was successful. Both male and female ESW increased the length of the femur and the number of ossicles, which produced significant therapeutic effects. However, femoral length and ossicle count reflect anatomical changes but not comprehensive biomechanical properties. Advanced methods like three-point bending tests and force-displacement analyses are necessary to evaluate bone strength and fracture resistance^45^. Our study provided a foundation for exploring ESW’s effects. Future research should incorporate biomechanical testing and micro-CT imaging to better understand the structural and mechanical interplay in GIOP models and the therapeutic mechanisms of ESW.

Disorders in liver function and immune regulation are closely associated with the development of osteoporosis^46–48^, as the liver plays a vital role in bone metabolism through proteins like vitamin D-binding protein and insulin-like growth factor-1^49,50^, while immune regulation impacts osteoclast and osteoblast activity via cytokines such as RANKL, TNF-α, and IL-6^51,52^. In our study, both male and female ESW treatments significantly improved liver and thymus indices, with female ESW also increasing spleen indices, suggesting a protective effect against osteoporosis through liver support and enhanced immunity. Female ESW demonstrated stronger immune-boosting effects compared to male ESW. The systemic effects of ESW may involve modulating inflammation and immune responses, as evidenced by its impact on cytokine levels and its potential to alleviate liver dysfunction. These findings highlight the interconnected roles of liver function and immune regulation in bone health and underscore the therapeutic potential of ESW in mitigating osteoporosis through systemic mechanisms. Further studies are needed to explore these pathways in depth and confirm the observed effects.

Bone metabolism disorders are the main cause of orthopedic diseases. Serum TRACP-5b is mainly derived from osteoclasts and indicates osteoclast activity. OT/BGP is mainly derived from osteoblasts and indicates osteoblast activity. BALP is secreted by osteoblasts to promote bone mineralization, and its content can reflect bone transformation^53^. What is exciting is that the female ESW can adjust three of the indicators back to normal levels, indicating that the female ESW can effectively regulate bone metabolism. In addition, OT/BGP levels in the MESWTG were significantly lower than those in FESWTG, suggesting potential sex-specific differences in osteoblast activity and bone metabolism. These findings may reflect the influence of sex-specific hormonal and molecular mechanisms on bone formation. For instance, estrogen has been shown to play a pivotal role in promoting osteoblast function^54^, which could partially explain the observed differences between male and female groups. In addition, we also found that male ESW significantly decreases the activity of osteoblasts and osteoclasts compared with normal levels, which raises potential concerns regarding its effects on bone homeostasis. The observed decrease in both bone formation and resorption may lead to an imbalance in bone remodeling, potentially compromising bone health over time^55^. For example, in one study, bone formation in male mice decreased significantly, while female mice exhibited an increase in bone resorption, which may be related to a deficiency in sex hormones^56^. While this effect may not immediately manifest in overt bone disorders, the long-term consequences on bone density and strength are important areas for future investigation. Further studies are needed to explore the mechanisms behind these changes in bone cell activity and to evaluate the clinical implications of male ESW use in bone health. It has been proved in animal and cellular levels that female ESW can accelerate the process of fracture healing^57^, of which mechanism may be related to the promotion of BMP and VEGF expression^58^.

Calcium and phosphorus metabolism is closely related to bone metabolism. The contents of Ca and P in serum are relatively balanced. Low Ca and high P will aggravate bone resorption and cause osteoporosis^59^. Although female and male ESW could not significantly increase the serum Ca level, they could increase it to a normal level. The female ESW significantly reduced the serum P level and maintain the normal calcium and phosphorus metabolism, while the effect of the male ESW was not ideal. Bone mineral deposition is closely related to the interaction of inorganic elements such as Mg, Cu and Zn. It has been reported that excessive Cu will interfere with bone metabolism and lead to a decrease in bone strength^60^, and a high Mg level can significantly inhibit the deposition of the mineral matrix in osteoblasts^61^. Both female and male ESW significantly restored the serum Mg level in osteoporotic mice, and female ESW also significantly restored the serum Cu level. In conclusion, there are great differences in the efficacy of female and male ESW in bone grafting, and the therapeutic effect of female ESW is obviously better than that of male ESW.

Our study provides a comprehensive comparison of female and male ESW in terms of composition and pharmacological effects. Female ESW demonstrated a greater abundance of bioactive components, including higher levels of small peptides, prostaglandins, and other metabolites, which contribute to its superior therapeutic effects. Specifically, female ESW exhibited significantly stronger anti-thrombotic and anti-osteoporotic effects, providing robust scientific evidence for its clinical preference.

The superior anti-osteoporotic effects of female ESW are mediated through its regulation of key biomarkers, such as TRACP-5b, OT/BGP, BALP, and serum inorganic elements (e.g., P, Mg, Cu). These findings suggest that female ESW effectively restores the balance between bone formation and resorption and improves calcium-phosphorus metabolism. The presence of enriched bioactive compounds may further support cellular repair and differentiation, potentially involving pathways like RANK/RANKL/OPG and Wnt/β-catenin. Studies have shown that the RANK/RANKL/OPG signaling pathway plays a crucial role in bone remodeling, regulating the balance between bone resorption and bone formation. Specifically, increased expression of RANKL promotes the differentiation and activity of osteoblasts, leading to the development of osteoporosis^62^. In addition, the Wnt/β-catenin signaling pathway plays a crucial role in bone formation. Activation of this pathway can promote the proliferation and differentiation of osteoblasts, thereby enhancing bone density. Studies have found that abnormalities in the Wnt signaling pathway may be closely related to the occurrence of osteoporosis. For example, downregulation of miR-146a has been found to inhibit the development of osteoporosis by modulating the Wnt/β-catenin signaling pathway^63^.

Although male ESW is less effective than female ESW, it still contains valuable bioactive components that could be utilized for active ingredient extraction, animal feed, and other applications. This opens avenues for resource optimization and warrants further research into its specific bioactive compounds and potential therapeutic applications.

## Conclusion

This study demonstrated that female ESW contains significantly higher levels of pharmacologically active compounds compared to males, indicating superior therapeutic effects in treating thrombosis and osteoporosis. Metabolomics identified 31 types of compounds in female and male ESW. The top three compounds in terms of relative percentage were lipids and lipid molecules, amino acids and their derivatives, and fatty acids. While the composition of the compounds was similar between females and males, their quantities differed. Eight pharmacologically active compounds, 15 small peptides, and 13 prostaglandins were found to be more abundant in female ESW than that in male ESW. These advantageous components in female ESW may serve as the material basis for their superiority in medicine compared to males, and further validation is required. Moreover, in vivo pharmacological experiments revealed the mechanisms behind the superiority of female ESW over male ESW. There were significant differences in the anti-osteoporotic effect between male and female ESW, with females exhibiting a clear advantage. This research is pioneering in its systematic comparison of male and female ESW using metabolomics and pharmacodynamics, filling a critical gap in understanding their medicinal properties. The superior efficacy of female ESW underscores its exclusive use in bone metabolic diseases, while also suggesting potential strategies for optimizing the utilization of male ESW resources such as animal feed additives. These results not only enhance our understanding of *Eupolyphaga sinensis* Walker but also pave the way for improved quality control standards and more effective therapeutic applications in traditional medicine.

## Materials and methods

### Experimental reagents

Acetonitrile and methanol were purchased from Merck (Darmstadt, Germany). Formic acid and 2-chlorophenylalanine were purchased from Thermo Fisher Scientific (Waltham, MA, USA). All reagents were of HPLC grade. Deionized water was obtained from a Milli-Q system (Millipore, USA).

K-carrageenan (S30561-25 g) and sodium citrate anticoagulant (R21531-100 ml) were purchased from Shanghai Yuanye Biotechnology Co., Ltd. (Shanghai, China). Carboxymethylcellulose sodium (CMC-NA) (RF1013) was purchased from Shanghai Ruiyong Biotechnology Co. Ltd. ELISA kits for prothrombin time (PT) (R01002), activated partial prothrombin time (APTT) (R01102) and fibrinogen (FIB) (R01302) were purchased from Shenzhen Redu Life Technology (Shenzheng, China). ELISA kits for plasminogen (PLG) (F10531-A), tissue-type plasminogen activator (t-PA) (F5561-A), plasminogen activator inhibitor (PAI) (F5106-A), 6-keto‐prostaglandin (6-K-PG) (F5069-A), thromboxane b2 (TXB2) (F5558-A), d-dimer (D2D) (F9951-A), osteocalcin/bone glutamate protein (OT/BGP) (F5590-A), bone alkaline phosphatase (BALP) (F7725-A) and tartrate-resistant acid phosphatase 5b (TRACP-5b) (F5447-A) were purchased from Feiya Biotechnology (Guangzhou, China). Total protein (A045-4-1), superoxide dismutase (SOD) (A001-1), malondialdehyde (MDA) (A003-1), calcium (C004-2), magnesium (C005), copper (E010-1-1), and zinc (E011-1-1) assay kits were purchased from Nanjing Jiancheng Biochemical Corporation (Nanjing, China). Paraformaldehyde solution (G1101-500 ml) was purchased from Wuhan Sevier Biotechnology Co., Ltd. (Wuhan, China); aspirin enteric-coated tablets were purchased from Bayer Healthcare Co., Ltd. (Leverkusen, Germany). The 0.9% sodium chloride solution was purchased from Shandong Qidu Pharmaceutical Co. Ltd. (Shandong, China). Dexamethasone sodium phosphate injection (1 ml: 5 mg) was purchased from Shanxi Ruicheng Kelong Veterinary Medicine Co., Ltd. (Shanxi, China). The blood phosphorus concentration assay kit (BC1655) was purchased from Solarbio (Beijing, China).

### Metabolomics-based investigation on the composition of female and male ESW

#### Sample collection, pre-treatment, and preparation

**Fig. 8:**
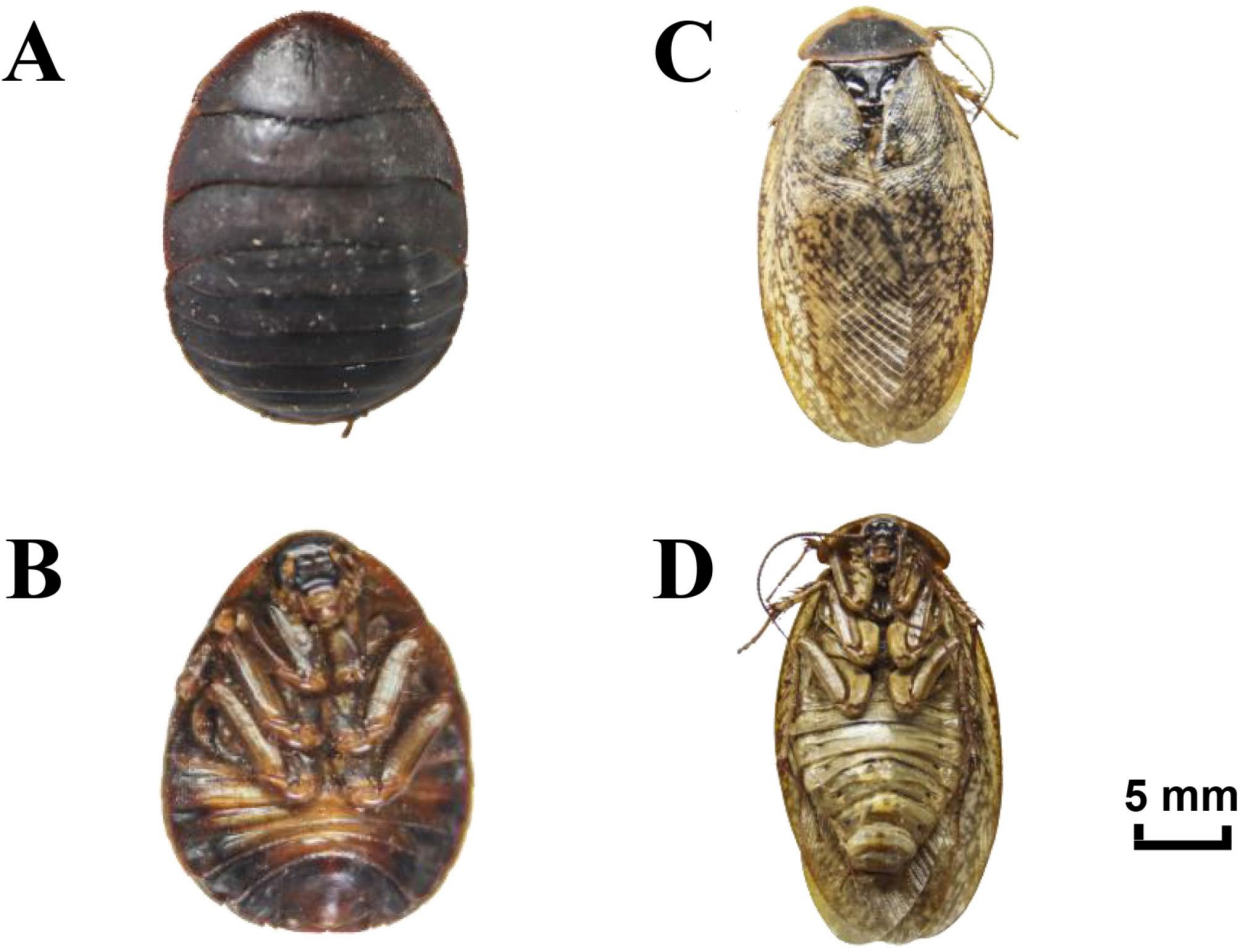
Adult *Eupolyphaga sinensis* Walker. The chest (**A**) and back (**B**) of female *Eupolyphaga sinensis* Walker; the chest (**C**) and back (**D**) of male *Eupolyphaga sinensis* Walker.

Samples of female and male *Eupolyphaga sinensis* Walker were collected from the Xinxing Tuyuan Professional Cooperative in Jiangsu, China (31°50′4.04″N, 119°46′51.03″E) at 7 months old, ensuring similar age and health status to minimize variability. They were fasted for three days before collection. Their overall appearance is shown in Fig. 8. They were identified as *Eupolyphaga sinensis* Walker by Professor Qiaosheng Guo at the Institute of Chinese Medicinal Materials of Nanjing Agricultural University. Vouchers for the sample were kept at Nanjing Agricultural University. The ESW was scalded to death by boiling water and then dried at 70 °C for 14 h^11^. After returning to room temperature, the samples were stored in a cool cabinet.

Sample preparation steps for metabolomics analysis: The specimens were pulverized to a fine powder (200 mesh), and 50 mg of the powder was transferred into a 2-mL centrifuge tube. Next, 500 µL of ice-cold 70% methanol, containing 2-chlorophenylalanine as an internal standard, was added to the tube. The mixture was vortexed for 30 s four times, then kept on ice for 15 min. It was centrifuged at 12,000 rpm and 4 °C for 10 min, after which 400 µL of the supernatant was transferred to a new tube. To the sediment, 500 µL of a 1:3 (v/v) ethyl acetate/methanol mixture was added, followed by shaking for 5 min, a 15-minute incubation on ice, and centrifugation at 12,000 rpm and 4 °C for 10 min. The supernatant (400 µL) was collected and combined with the previous supernatant. The combined solution was then concentrated. Finally, 100 µL of 70% methanol in water was added, and the mixture was sonicated for 3 min before centrifuging at 12,000 rpm and 4 °C for 3 min. The supernatant was collected for LC-MS/MS analysis.

#### Chromatography and mass spectrometry conditions

The instrument conditions used were previously reported^64^. The mobile phase consisted of 0.1% formic acid in water (A) and 0.1% formic acid in acetonitrile (B). The linear gradient elution conditions were as follows: 0 ~ 11 min, 5% B; 11 ~ 12.1 min, 90% B; 12.1 ~ 14 min, 5% B. For MS conditions, an electrospray ionization source in both positive (ESI+) and negative (ESI-) modes was selected.

#### Identification and overall analysis of components

The raw data from the mass spectrometer were converted to mzML format using ProteoWizard. Peak extraction, alignment, and retention time correction were performed using the XCMS program. The ‘SVR’ method was then used to correct the peak area. The peaks were calibrated again, and component identification information was obtained by searching self-built databases, integrating public libraries, and metDNA.

This study focused on analyzing ingredients with secondary identification information and determining the types, quantities, and relative percentages of these ingredients. The top 10 components of peak area (expression) in male and female ESW with a score of 0.8 or higher were specifically highlighted.

#### Screening of differential components between female and male ESW

Based on the variable importance in projection (VIP) value of the orthogonal partial least squares discriminant analysis (OPLS-DA) model, the differential components were initially screened. These components were further screened in combination with the *p* value and the fold change (FC) value by univariate analysis. The types and quantities of the differential components are counted and displayed by a three-dimensional histogram. The screening conditions were as follows: (1) VIP ≥ 1; (2) *p* value<0.05; and (3) FC ≥ 2 or FC ≤ 0.5.

#### Bioinformatics analysis of differential components

The Kyoto Encyclopedia of Genes and Genomes (KEGG) database^65^ was used to annotate the differential components screened in the ESI + and ESI- modes and to perform enrichment analysis. The top 20 pathways are displayed in a bubble chart.

#### Screening of dominant components in the female ESW

Only the components with toxicity and pharmacological activity were screened from the differential components with down-regulated expression in male EWS, and the screening conditions were (1)-(5). In addition, small peptides and prostaglandins that female ESW have advantages over male ESW were screened, and the screening conditions were (1)-(2). The screening conditions were as follows: (1) VIP ≥ 1; (2) peak area ≥ 1 × 1,000,000 cps (counts/second) in female ESW samples; (3) components have CAS ID; (4) chemical information search (https://www.chemsrc.com) and literature review showing pharmacological activity; and (5) qualitative score (score) ≥ 0.7.

### The anti-thrombotic effect of female and male ESW

#### Experimental animals

Five-week-old male ICR mice (30 ± 2 g) were purchased from Zhejiang Weitong Lihua Experimental Animal Technology Co., Ltd. [licence number SCXK (Zhejiang) 2019-0001]. They were kept in the barrier system (20 ± 2 °C) of the Animal Experiment Center of Nanjing Agricultural University. The adaptive feeding experiment was conducted for 3 days, after which the formal experiment started. The experimental procedures and animal care conditions were approved by the Experimental Animal Welfare Ethics Committee of Nanjing Agricultural University (NJAU. No. 20210810123). The experimental protocol was in accordance with the Regulations of Experimental Animal Administration issued by the State Committee of Science and Technology of the People’s Republic of China. Additionally, this study was reported in accordance with Animal Research: Reporting of In Vivo Experiments (ARRIVE) guidelines (https://arriveguidelines.org).

#### Sample processing

Adult female and male ESW were scalded to death by boiling water and then dried at 70 °C for 14 h. After returning to room temperature, the samples were stored in a cool cabinet. Samples were pulverized (200 mush) before use.

#### Construction of the tail thrombosis model

Referring to the literature^66^, k-carrageenan was dissolved in deionized water at 60 °C to make a solution (0.5 g/100 ml). One hour after intragastric administration on the seventh day, except for the normal control group, the other groups were intraperitoneally injected with 0.2 ml/10 g (100 mg/kg) k-carrageenan to establish the models. The normal control group was intraperitoneally injected with the same volume of normal saline. The mice were fasted for 12 h before dissection. After 24 h of modelling, blood was taken from the eyeballs. Finally, all the mice were euthanized by cervical dislocation.

#### Grouping and administration

After adaptive feeding, the mice were randomly divided into 5 groups: the normal control group (NCG), model group (MG), positive drug treatment group (PDTG), female ESW treatment group (FESWTG) and male ESW treatment group (MESWTG), with 10 mice in each group.

The common clinical dose of ESW is 6 g^67^. The medium dose was calculated according to the clinical dose and the conversion formula of body surface area^68^, and the low and high doses were 0.5 times and 2 times of the medium dose, respectively. According to the results of the preliminary experiment, the high dose (1.56 g/kg) was ultimately selected for administration. The drug administration scheme was as follows: FESWTG was orally administered once daily with suspension (the female ESW sample powder mixed with 1% CMC-NA) at a dose of 1.56 g/kg; MESWTG was orally administered once daily with suspension (the male ESW sample powder mixed with 1% CMC-NA) at a dose of 1.56 g/kg; PDTG was orally administered once daily with aspirin enteric-coated tablets at a dose of 100 mg/kg; and the other groups were orally administered with the same amount of 1% CMC-NA. This administration process was continued for 7 days.

#### Determination of the tail thrombosis rate

After the mice were dissected on ice, the length of the thrombus covering the tail was measured, and the percentage of tail thrombosis was calculated as the length of thrombus covering the tail (cm)/total length of the tail (cm) × 100%.

#### Determination of organ indices

After the mice were dissected on ice, the liver, spleen and thymus were quickly collected, washed with ice-cold physiological saline, blotted with sterilized filter paper, and then weighed for organ coefficient by organ weight (g)/body weight (g) × 100%.

#### Observation of pathological tissue slices

The right femurs of the mice were fixed in 4% paraformaldehyde, dehydrated, and embedded in paraffin. The paraffin-embedded tissues were sectioned, stained with hematoxylin and eosin (H&E), and observed under a light microscope (Leica, DMC5400, Germany).

#### Determination of MDA content and SOD activity in liver and tail tissues

The MDA and SOD content in mouse liver and tail pathological tissues were measured by assay kits according to the manufacturer’s instructions.

#### Determination of plasma biochemical indices

The contents of FIB, PT, APTT, PLG, t-PA, PAI, D2D, 6-K-PG, and TXB2 in blood plasma were determined according to the kit instructions and the ratio of TXB2/6-K-PG was calculated.

### The anti-osteoporotic effect of female and male ESW

#### Experimental animals

Eight-week-old male ICR mice (35 ± 2 g) were purchased from Zhejiang Weitong Lihua Experimental Animal Technology Co., Ltd. [licence number SCXK (Zhejiang) 2019-0001]. The feeding conditions were the same as those described in section “ The anti-thrombotic effect of female and male ESW”. The experimental animal welfare and ethics review number is NJAU. No. 20,210,630,100. The experimental protocol was in accordance with the Regulations of Experimental Animal Administration issued by the State Committee of Science and Technology of the People’s Republic of China. Additionally, this study was reported in accordance with Animal Research: Reporting of In Vivo Experiments (ARRIVE) guidelines (https://arriveguidelines.org).

#### Construction of the osteoporosis model

According to previously reported methods^69^, except for the normal control group, the other groups were intramuscularly (gluteal) injected with 0.01 ml/10 g (5 mg/kg) dexamethasone sodium phosphate injection (1 ml: 5 mg) for 4 times a week to establish the models. The modelling process lasted for 4 weeks. The normal control group was intramuscularly (gluteal) injected with the same volume of normal saline. The mice were fasted for 12 h before dissection. After the last administration, blood was taken from the eyeballs. Finally, all the mice were euthanized by cervical dislocation.

#### Grouping and administration

After adaptive feeding, the mice were randomly divided into 4 groups: the normal control group (NCG), model group, female ESW treatment group (FESWTG) and male ESW treatment group (MESWTG), with 10 mice in each group.

The drug administration scheme was as follows: FESWTG was orally administered once daily with a suspension (the female ESW sample powder mixed with 1% CMC-NA) at a dose of 1.56 g/kg; MESWTG was orally administered once daily with a suspension (the male ESW sample powder mixed with 1% CMC-NA) at a dose of 1.56 g/kg; and the other groups were orally administered with the same amount of 1% CMC-NA. This administration process was continued for 4 weeks.

#### Determination of femoral biomechanical parameters

After the mice were dissected on ice, the right femur was taken and the attached muscle tissue was removed. The femur was accurately weighed, and the length and diameter were accurately measured using a Vernier calliper.

#### Determination of organ indices

After the mice were dissected on ice, the kidney, liver, spleen and thymus were quickly collected, washed with ice-cold physiological saline, blotted with sterilized filter paper, and then weighed for organ coefficient by organ weight (g)/body weight (g) × 100%.

#### Observation of pathological tissue slices

The right femur was fixed in 4% paraformaldehyde for more than 24 h, followed by decalcification, dehydration, clearing, paraffin infiltration and embedding. The paraffin-embedded tissues (mid-diaphysis) were sectioned, stained with hematoxylin and eosin (H&E), and observed under a light microscope (Leica, DMC5400, Germany).

#### Determination of plasma biochemical indices

The contents of BALP, TRACP-5b, OT/BGP, Ca, P, Zn, Cu and Mg in blood serum were determined by assay kits according to the manufacturer’s instructions.

### Data processing and multivariate statistical analysis

Statistical analyses were performed using one-way ANOVA with Multiple comparisons between groups with GraphPad Prism 9.5.0 software. Significant differences are indicated as follows: ∗*p* < 0.05; ∗∗*p* < 0.01; ∗∗∗*p* < 0.001; ∗∗∗∗*p* < 0.0001; ns, not significant. As for metabolomics data, principal component analysis (PCA), OPLS-DA, Student’s t-test and FC analysis were carried out by R (base package) 3.5.0. Pathway analysis was performed using MetaboAnalyst 5.0 (https://www.metaboanalyst.ca/home.xhtml).

## Electronic supplementary material

Below is the link to the electronic supplementary material.

Supplementary Material 1

Supplementary Material 2

## Data Availability

Data is provided within the manuscript or supplementary information files.

## Abbreviations

ESW: *Eupolyphaga sinensis* Walker
CMC-NA: Carboxymethylcellulose sodium
TIC: Total ion chromatogram
ESI+: Positive mode
ESI−: Negative mode
QC: Quality control
PCA: Principal component analysis
VIP: Variable importance in projection
OPLS-DA: Orthogonal partial least squares discriminant analysis
FC: Fold change
KEGG: Kyoto encyclopedia of genes and genomes
ICR: Institute of cancer research
NCG: Normal control group
MG: Model group
PDTG: Positive drug treatment group
FESWTG: Female ESW treatment group
MESWTG: Male ESW treatment group
GIOP: Glucocorticoid-induced osteoporosis
