# Supplementary material for: Enhanced therapeutic efficacy of Eupolyphaga sinensis Walker in females through sex-specific metabolomic-pharmacodynamic divergence
Source: Sci Rep. 2025 Feb 19;15:6032. doi: 10.1038/s41598-025-90100-5 (PMC11839932; doi:10.1038/s41598-025-90100-5)
Supplement: Supplementary file 1 — Supplementary Material 1 [file 41598_2025_90100_MOESM1_ESM.docx]

***Supplementary Material***

**1 Supplementary Data**

**Dataset S1** Differential components annotated by KEGG.

**2 Supplementary Tables**

**Table S1** Types, quantities, and relative percentage content of components in female and male *Eupolyphaga sinensis* Walker.

| No. | Classification | ESI+ | | | ESI- | | |
| --- | --- | --- | --- | --- | --- | --- | --- |
|  |  | Quantity | Female (%) | Male (%) | Quantity | Female (%) | Male (%) |
| 1 | Lipids and lipid-like molecules | 1069 | 17.04 | 17.24 | 165 | 10.67 | 9.55 |
| 2 | Organoheterocyclic components | 403 | 11.28 | 8.60 | 81 | 1.79 | 2.22 |
| 3 | Benzene and substituted derivatives | 355 | 10.97 | 8.29 | 85 | 2.28 | 1.73 |
| 4 | Organic acid and its derivatives | 272 | 6.26 | 5.57 | 145 | 6.81 | 5.18 |
| 5 | Organic oxygen components | 137 | 2.49 | 2.48 | 40 | 0.41 | 0.66 |
| 6 | Amino acids and its metabolomics | 105 | 10.95 | 10.32 | 41 | 2.68 | 2.53 |
| 7 | Organic nitrogen components | 80 | 2.47 | 2.32 | 1 | 0.08 | 0.09 |
| 8 | Alkaloids and derivatives | 65 | 1.60 | 0.92 | 12 | 0.20 | 0.10 |
| 9 | Phenylpropanoids and polyketides | 64 | 0.98 | 2.02 | 41 | 0.82 | 0.72 |
| 10 | Nucleosides, nucleotides, and analogues | 58 | 3.20 | 4.08 | 25 | 1.17 | 1.57 |
| 11 | Alcohol and amines | 37 | 2.90 | 1.01 | 4 | 0.07 | 0.10 |
| 12 | Fatty acid | 36 | 5.40 | 13.10 | 11 | 29.30 | 22.05 |
| 13 | Glycerophospholipids | 21 | 3.36 | 3.74 | 11 | 0.75 | 0.66 |
| 14 | Oxidized lipids | 18 | 0.29 | 0.13 | 16 | 0.34 | 0.45 |
| 15 | Hormones and related compunds | 16 | 0.93 | 0.27 | 7 | 0.04 | 0.01 |
| 16 | Aldehyde, Ketones, Esters | 16 | 0.20 | 0.16 | 5 | 0.24 | 0.19 |
| 17 | Organosulfur components | 13 | 0.59 | 0.60 | 1 | 0.03 | 0.05 |
| 18 | Phenolic acids | 12 | 0.17 | 0.15 | 20 | 0.77 | 0.62 |
| 19 | Lignans, neolignans, coumarins and related components | 11 | 0.08 | 0.07 | 3 | 0.08 | 0.09 |
| 20 | Hydrocarbon and its derivatives | 10 | 0.05 | 0.06 | 1 | 0.01 | 0.01 |
| 21 | CoEnzyme and vitamins | 8 | 0.36 | 0.26 | 4 | 0.05 | 0.06 |
| 22 | Glycerolipids | 8 | 0.04 | 0.02 | 1 | 0.02 | 0.01 |
| 23 | Tryptamines, Cholines, Pigments | 6 | 1.21 | 0.29 | 0 | 0.00 | 0.00 |
| 24 | Carboxylic acids and derivatives | 6 | 0.15 | 0.17 | 6 | 0.19 | 0.32 |
| 25 | Organohalogen components | 5 | 0.21 | 0.23 | 0 | 0.00 | 0.00 |
| 26 | Bile acids | 5 | 0.03 | 0.02 | 8 | 0.25 | 0.11 |
| 27 | Sphingomyelin | 4 | 0.06 | 0.01 | 0 | 0.00 | 0.00 |
| 28 | Flavonoids | 4 | 0.02 | 0.02 | 3 | 0.00 | 0.00 |
| 29 | Terpenoids | 3 | 0.01 | 0.01 | 0 | 0.00 | 0.00 |
| 30 | Organometallic components | 1 | 0.25 | 0.24 | 0 | 0.00 | 0.00 |
| 31 | Organic 1, 3-dipolar components | 1 | 0.00 | 0.00 | 0 | 0.00 | 0.00 |
| 32 | Others | 746 | 16.45 | 17.59 | 79 | 40.93 | 50.93 |
|  | Total | 3595 | 100.00 | 100.00 | 816 | 100.00 | 100.00 |

ESI+, positive mode; ESI-, negative mode.

**Table S2** Types and quantities of different components between female and male *Eupolyphaga sinensis* Walker.

| No. | Classification | Quantity | |
| --- | --- | --- | --- |
|  |  | ESI+ | ESI- |
| 1 | Lipids and lipid-like molecules | 503 | 90 |
| 2 | Organoheterocyclic components | 239 | 30 |
| 3 | Benzene and substituted derivatives | 196 | 51 |
| 4 | Organic acids and derivatives | 164 | 74 |
| 5 | Organic oxygen components | 72 | 24 |
| 6 | Amino acids and derivatives | 56 | 17 |
| 7 | Organic nitrogen components | 42 | 0 |
| 8 | Alkaloids and derivatives | 41 | 8 |
| 9 | Phenylpropanoids and polyketides | 39 | 26 |
| 10 | Nucleosides, nucleotides, and analogues | 32 | 7 |
| 11 | Fatty acid | 26 | 6 |
| 12 | Alcohol and amines | 25 | 2 |
| 13 | Aldehyde, Ketones, Esters | 12 | 3 |
| 14 | Hormones and hormone related compunds | 11 | 4 |
| 15 | Oxidized lipids | 10 | 10 |
| 16 | Phenolic acids | 9 | 10 |
| 17 | Glycerophospholipids | 7 | 5 |
| 18 | Lignans, neolignans, Coumarins and related components | 6 | 2 |
| 19 | Organosulfur components | 5 | 0 |
| 20 | CoEnzyme and vitamins | 5 | 3 |
| 21 | Tryptamines, Cholines, Pigments | 4 | 0 |
| 22 | Sphingomyelin | 4 | 0 |
| 23 | Hydrocarbons and its derivatives | 4 | 1 |
| 24 | Bile acids | 3 | 3 |
| 25 | Terpenoids | 3 | 0 |
| 26 | Glycerolipids | 3 | 1 |
| 27 | Flavonoids | 2 | 3 |
| 28 | Carboxylic acids and derivatives | 2 | 2 |
| 29 | Organohalogen components | 1 | 0 |
| 30 | Organic 1, 3-dipolar components | 1 | 0 |
| 31 | Others | 425 | 32 |
|  | Total | 1952 | 414 |

ESI+, positive mode; ESI-, negative mode.

**Table S3** Statistics of different components.

| Mode | Group name | Total significant metabolites | Down-regulated | Up-regulated |
| --- | --- | --- | --- | --- |
| ESI+ | Female vs male ESW | 1952 | 1134 | 818 |
| ESI- | Female vs male ESW | 414 | 229 | 185 |

ESW, *Eupolyphaga sinensis* Walker. ESI+, positive mode; ESI-, negative mode.

**2 Supplementary Figures**


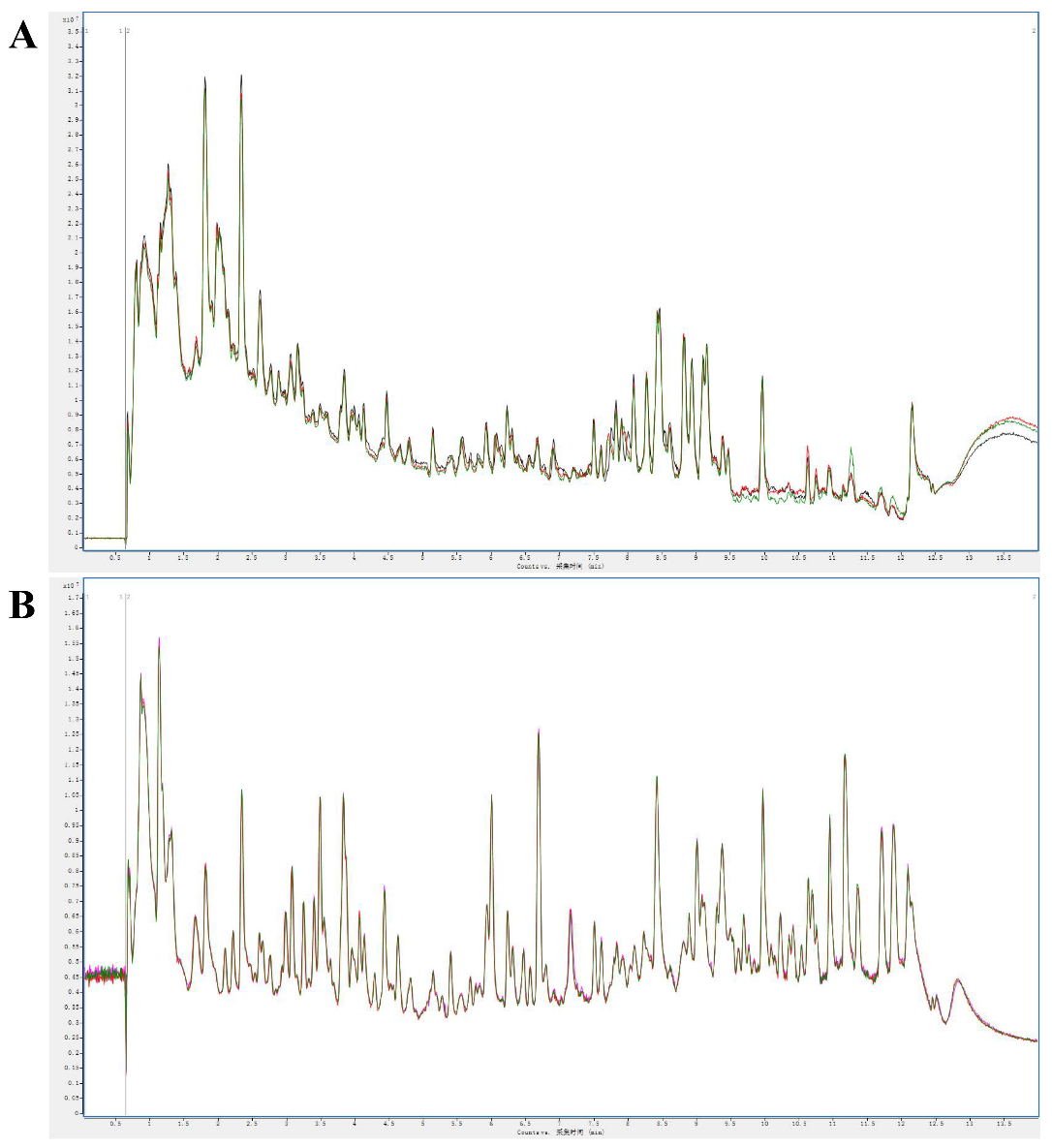


**Figure S1** TIC overlap plot for QC samples by mass spectrometry detection.

(A) Positive mode; (B) Negative mode. TIC, total ion chromatogram; QC, quality control.
